# Differential migration in Chesapeake Bay striped bass

**DOI:** 10.1371/journal.pone.0233103

**Published:** 2020-05-14

**Authors:** David H. Secor, Michael H. P. O’Brien, Benjamin I. Gahagan, J. Carter Watterson, Dewayne A. Fox

**Affiliations:** 1 Chesapeake Biological Laboratory, University of Maryland Center for Environmental Science, Solomons, Maryland, United States of America; 2 Massachusetts Division of Marine Fisheries, Gloucester, Massachusetts, United States of America; 3 United States Navy, Naval Facilities Engineering Command Atlantic, Norfolk, Virginia, United States of America; 4 Delaware State University, College of Agriculture, Science, and Technology, Dover, Delaware, United States of America; Havforskningsinstituttet, NORWAY

## Abstract

Differential migration—increased migration propensity with increasing individual size—is common in migratory species. Like other forms of partial migration, it provides spatial buffering against regional differences in habitat quality and sources of mortality. We investigated differential migration and its consequences to survival and reproductive patterns in striped bass, a species with well-known plasticity in migration behaviors. A size-stratified sample of Potomac River (Chesapeake Bay) *Morone saxatilis* striped bass was implanted with acoustic transmitters and their subsequent coastal shelf migrations recorded over a 4-yr period by telemetry receivers throughout the Mid-Atlantic Bight and Southern New England. A generalized linear mixed model predicted that ≥ 50% of both males and females depart the Chesapeake Bay at large sizes >80 cm total length. Egressing striped bass exited through both the Chesapeake Bay mouth and Delaware Bay (via the Chesapeake and Delaware Canal), favoring the former. All large fish migrated to Massachusetts shelf waters and in subsequent years repeatedly returned to regions within Massachusetts and Cape Cod Bays. Within this dominant behavior, minority behaviors included straying, skipped spawning, and residency by large individuals (those expected to migrate). Analysis of the last day of transmission indicated that small resident striped bass experienced nearly 2-fold higher loss rates (70% yr^-1^) than coastal shelf emigrants (37% yr^-1^). The study confirmed expectations for a threshold size at emigration and different mortality levels between Chesapeake Bay (resident) and ocean (migratory) population contingents; and supported the central premise of the current assessment and management framework of a two-contingent population: smaller Chesapeake Bay residents and a larger ocean contingent. An improved understanding of differential migration thus affords an opportunity to specify stock assessments according to different population sub-components, and tailor reference points and control rules between regions and fishing stakeholder groups.

## Introduction

Partial migration, the presence of resident and migratory ecotypes, influences how populations and metapopulations respond to regionally varying environmental and anthropogenic forcing, and can stabilize population dynamics [1–3]. The traditional view that migrations by fishes are uniform seasonal behaviors [4, 5] has been challenged by mounting evidence for the prevalence of plasticity in migration behaviors. Partial migration, well known for taxa such as salmons and chars, is now known to be common in bony fishes, elasmobranchs, and indeed vertebrate taxa writ large [5–7]. Our improved understanding of migration behavior gives rise to a new and important challenge: building partial migration behaviors into conservation and stewardship frameworks that promote sustainability and resilience.

In partial migration systems, whether to migrate or not is often dependent on size–known as differential migration [8]. Size shapes the outcome of migration through its influence on swimming performance, forage demand, predation risk, and reproductive provisioning [6, 9, 10]. For instance, reduced cost of movement with size could favor increased ranges in larger adults [9] as is commonly observed among diverse taxa including cods, flatfishes, snappers and drums [6]. Still, in other species such as Pacific bluefin tuna *Thunnus orientalis*, juveniles exhibit increased levels of transoceanic migrations [11]. Farther ranging individuals will encounter differing environmental and anthropogenic conditions than those smaller (or larger) individuals that stay near home, resulting in more nuanced population outcomes than would be expected were seasonal migrations are uniform. Such outcomes can relate to differential survival, growth, and reproduction.

The importance of relating size-dependent migration behaviors to environments encountered by migratory and resident individuals is reflected in the problem of assessing and managing estuarine and shelf fisheries for Atlantic striped bass *Morone saxatilis*. Chesapeake Bay population striped bass are large (maxima: 40 kg; 150 cm total length (TL); 33 years; DHS, pers. obs), relatively late-maturing (female and male full maturation: 8 and 4 years, respectively [12]), and migratory within NW Atlantic shelf and estuarine environments, spawning in tidal freshwater. After spawning, adults migrate north to New England waters in spring and summer and then migrate south during late fall and winter to South Atlantic Bight shelf waters as far south as North Carolina. Owing to their extent and productivity, natal habitats in the Chesapeake Bay contribute the most recruits to the US NW Atlantic [12–14], supporting both valuable commercial fisheries (2017: $4.6 million; 2.1 10^3^ tons [12]) and recreational fisheries (top-ranked US marine recreational fishery: 2017: 17.2 10^3^ tons [12]). Recreational fisheries predominate in shelf waters (although also occurring in the Chesapeake Bay) and target larger individuals. In comparison, most commercial landings are from the Chesapeake Bay and comprise smaller individuals. A recent assessment indicated that total mortality rates in the Chesapeake Bay were substantially higher than those for the shelf stock [12]. Managing US regional allocations of striped bass thus depends on predicting the size and age at which striped bass move from their natal estuary to shelf environments, given each region’s unique size-dependent removal patterns [12].

Striped bass show high plasticity in migration behaviors [15–17]. Ocean incidence varies by sex and increases with size and age [16, 18, 19], although high abundance of smaller immature striped bass have been noted in near shelf waters [13, 20–22]. Conversely, a minority of large adult striped bass never migrate to shelf waters [23]. Analyzing striped bass tagged in the Potomac River (Chesapeake Bay), Kohlenstein [24] advanced the earliest hypothesis related to differential migration: that young striped bass remain in or near the tributary in which they were spawned for two or three years. After this age, a substantial proportion (~50%) of immature females emigrate from the Bay, while the remaining immature and mature males remain in the Bay throughout their lives. In a Bayesian framework applied to conventional tagging data (n = 56 ocean returns), Dorazio et al. [25] contrasted patterns of likely size-specific egress by Chesapeake Bay and Hudson River striped bass and predicted that ≥50% (TL_50_) of striped bass egress at sizes >80 cm TL, regardless of sex. Based on otolith tracer analysis of 82 females and 40 males, Secor and Piccoli [23] also detected a trend of increasing egress with length, with approximately 40% predicted to egress at >80 cm TL. In contrast to Dorazio et al.’s prediction of full egress at >100 cm TL, Secor and Piccoli predicted that a small minority of these larger striped bass resided in the Chesapeake Bay.

A unique and timely opportunity availed itself to leverage extensive coastal biotelemetry assets only recently deployed within the US NW Atlantic to understand the coastal migrations of striped bass through cooperative data sharing. During the period 2014–2018, we tracked migrations of size-stratified groups of 75 (spring 2014), and 25 (fall 2014) Potomac River striped bass implanted with acoustic transmitters within the Chesapeake and throughout the US NW Atlantic Coast. Over 4 10^5^ telemetry detections were compiled to address a set of hypotheses related to: seasonal migration patterns and degree of residency within the Chesapeake Bay, and patterns of inferred mortality, migration routes, straying and skipped spawning. These included expected seasonal shelf migration patterns, higher mortality in Chesapeake Bay residents, and incidence of straying and skipped spawning in both resident and migratory contingents [23, 26].

## Materials and methods

### Study site and experimental fish

This study required animal research. Approval was received by the University of Maryland Center for Environmental Science IACUC: #F-CBL-14-05. Prior to surgerical implantation of transmitters, an anaesthetic was applied of 60 mg L-1 of tricaine methanesulfonate and 30 mg L-1of quinaldine sulfate.

**Fig 1:**
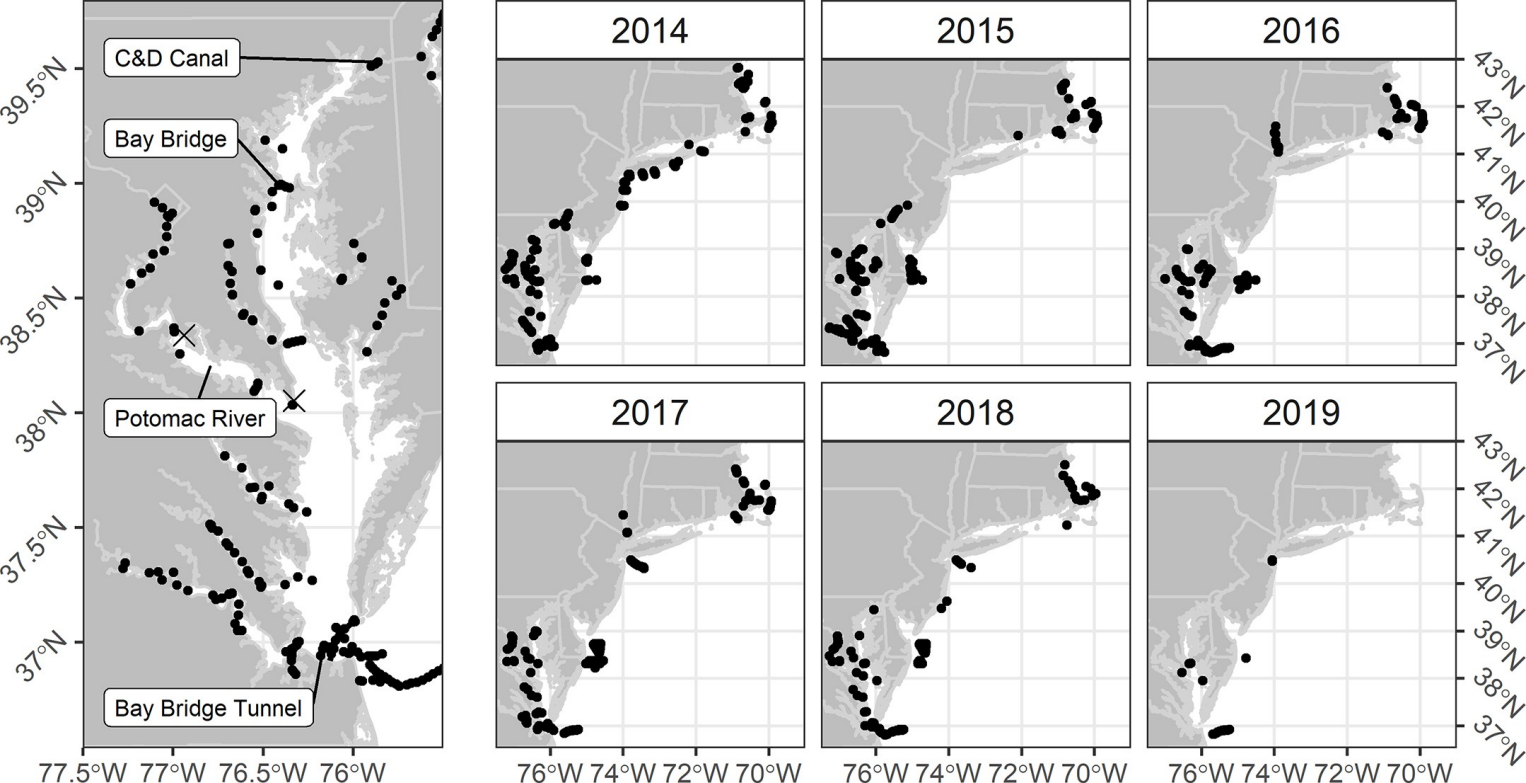
Telemetry receivers deployed in Mid-Atlantic and southern New England estuarine and shelf regions. Receivers shown as filled circles, where Potomac River striped bass have been detected. Locations of tagging locations shown by X symbols. Note disappearance of NY Bight receivers 2015–2016 and increased DelMarVa shelf receiver arrays in 2015–2018.

Deployments of acoustic telemetry arrays in US NW Atlantic shelf waters is unprecedented (Fig 1), supporting evaluations of broad scale coastal migrations by Chesapeake striped bass from 2014–2019. Here the Atlantic Coastal Telemetry (ACT) Network (www.theactnetwork.com), an online portal that allows distributed telemetry investigators to share telemetry detection data, aided us. Receiver arrays maintained by ACT network scientists also occur in major estuaries (Chesapeake Bay mainstem and tributaries, and Delaware Bay and Hudson River estuaries) where striped bass forage and reproduce (Fig 1). We augmented existing assets, deploying and maintaining receivers (VEMCO VR2W ©) just below spawning habitats in the Potomac River, the lower Potomac River and two arrays across the mainstem of the Chesapeake Bay, just north of the Potomac River and below the Chesapeake Bay Bridge at Kent Island (Fig 1). These Chesapeake Bay receivers were deployed over the analysis period, 2014–2018, and visited for maintenance and data downloading once every 2–3 months; those in tributaries were removed during winter months to avoid loss owing to freezing events.

**Fig 2:**
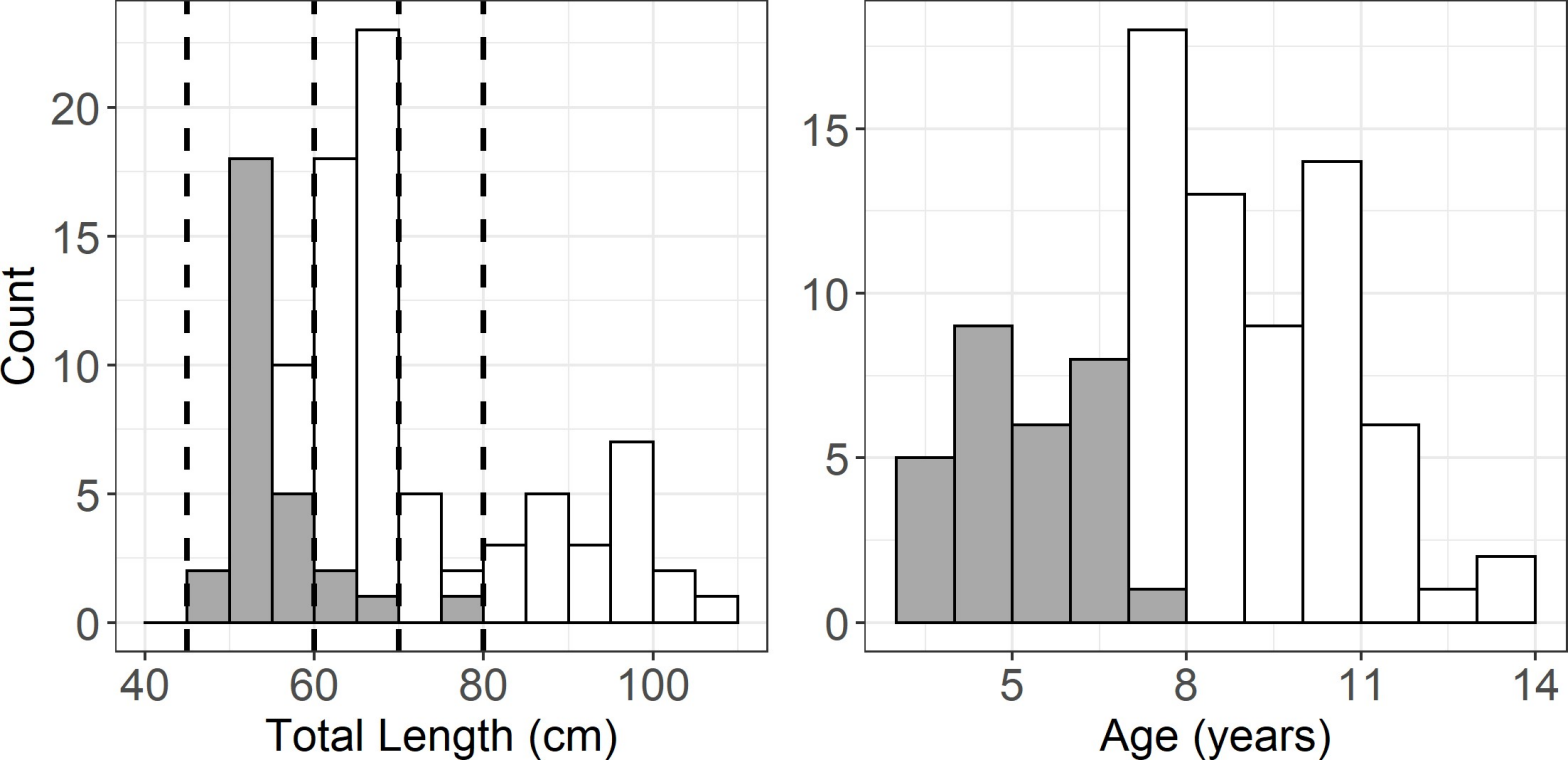
Size (total length, TL) and age distributions of experimental striped bass. Striped bass collected and released during spring in mid-estuary gillnets (open bars) and fall in a down-estuary pound net (filled bars). Dashed lines indicate targeted size strata.

Four size-classes were targeted that emphasized the size range over which differential migration was expected to change most rapidly. The sample size, 100, represented a compromise between cost and statistical power of inferences expected for this sample size based on binomial likelihood. Targeted size strata were 45–60, 61–70, 71–80, and >80 cm TL. The smallest size class, which is not well represented in spawning runs, was sampled in the fall and represented the presumed resident group [27]; larger size classes were drawn from the spring spawning run. Smaller-interval medium size classes focused on sizes where intermediate levels of emigration were expected. We contracted a commercial fishers to procure spring run striped bass through gill net sampling in the middle-Potomac River Fig 1), releasing fish from 30 March to 11 April 2014. Similarly, we contracted a pound net fisher near the mouth of the Potomac (Fig 1) to obtain and release smaller resident fish on 30 October 2014. Striped bass receiving transmitters ranged from 56–106 cm and 45–77 cm TL in the spring and fall releases, respectively (Fig 2). Despite efforts to uniformly sample among targeted size strata, higher numbers occurred for the 45–60 cm (n = 29) and 61–70 cm (n = 43) strata in comparison to the 70–80 cm (n = 7) and >80 cm (n = 21) strata. Fish >80 cm were predominately female (13 females, 4 males, 4 unidentified). Ages (methods presented below) were more normally distributed than lengths, ranging between 3 and 13 years with a mode at 7 years (Fig 2). Fall-released fish predominately comprised mature males (maturation between 2–4 years) and immature females (50% maturation at 6 years) [12]. Fish >80 cm TL were assumed to be fully mature [12].

Following their removal from gill nets or pound nets, fish were held in an 8-m^3^ floating pen to ensure their recovery from harvest and to minimize stress prior to surgery. We implanted coded telemetry transmitters (VEMCO; model V16-4H-S256; 6.8 cm, 10 g, 3.0-year expected battery life, random ping rate 90–120 s) following previous procedures [28, 29] under an approved protocol by the University of Maryland Center for Environmental Science IACUC (#F-CBL-14-05). Striped bass were anesthetized in a solution of 60 mg L^-1^ of tricaine methanesulfonate and 30 mg L^-1^of quinaldine sulfate. Following sedation, fish were measured for length and weight, and the transmitter implanted through a 25-mm midline incision located anterior to the pelvic girdle. Gonads were inspected through the incision for sex determination. The incision was closed using sterile, absorbable surgical monofilament. Fish were released following regaining equilibrium and locomotion in a large recovery tank. In previous experimental trials conducted on Patuxent River striped bass [28], this procedure resulted in no detectable long-term (3 month) changes to the stress or health of individuals. Transmitters were labeled to encourage the reporting of recapture information following harvest. External T-bar tags were attached, with a label requesting release and providing contact information (this resulted in three responses by fishers). Scales were collected during the surgical procedure and retained for age determination (Fig 2). Scales were pressed between two petrographic slides and annuli interpreted according to a standard protocol [30] under a dissecting microscope. Scales generally provide valid ages until 10 years of age [30, 31].

### Differential mortality

Attrition of tagged fish occurred over the four years after release, and by April 2018 only 12 individuals (9 spring-; 3 fall-released) were accounted for through detections. Therefore, analyses were limited to March 2014 –February 2018. The last detection date for each individual represented their period at large (alive); subsequent to this date we assumed their loss from the sample (death). Individuals were summed across all dates that they were at large. Sums were log-transformed and regressed against days at large (date) for spring- and fall-release groups separately to estimate daily instantaneous loss rates. All analyses were conducted in R, version 3.6.3 [32].

### Differential migration

The effect of size (total length, weight) and age at emigration variables were estimated through separate generalized linear mixed models (GLMM). Each GLMM was fit using a binomial distribution and logistic link function, with the full model including a term to block for the influence of year, an interaction between the variable and year, and a random effect of individual fish. Threshold values of size and age were considered to be the 50^th^ percentile values (TL_50_, Wt_50_, and Age_50_) of the predicted GLMM response. Year-specific 50^th^ percentile values were calculated by dividing the negative estimate of each year’s intercept by its estimated slope while holding the random effect of individual fish constant.

$${\left(\mathrm{TL},\;\mathrm{Wt},\;\mathrm{Age}\right)}_{\mathrm{yr},50}=‐\frac{{\mathrm{Intercept}}_{\mathrm{yr}}}{{\mathrm{Slope}}_{\mathrm{yr}}}$$

Threshold sizes and age at migration were compared between years using likelihood ratio tests of each variable’s nested GLMMs, where a significant interaction term implied a significant influence of year. Overall 50^th^ percentile values were calculated by averaging the estimated parameters of each year and calculating a threshold value from the pooled estimate. Bootstrapped 95% confidence intervals were calculated for each threshold value. Analyses were conducted using the *lme4* package, version 1.1–21, in R, version 3.6.3 [33].

Prior to model fitting, fish analyzed for emigration were censored to include only those detected after August in a given year; it was assumed that coastal migration behavior would manifest within 3 months of the April-May spawning season. Years were demarcated as starting prior to the spawning season on March 1 of each year: 2014, March 1 2014—February 28, 2015; 2015, March 1, 2015 –February 29, 2016; 2016, March 1, 2016 –February 28, 2017; and 2017, March 1, 2017 –February 28, 2018. For each fish meeting this convention, receiver detections were classified as either ocean or Chesapeake Bay (bounded by the C&D Canal and Bay Bridge Tunnel (Fig 1)). For age models, age-at-release was advanced in accordance to the year of comparison. Because no such adjustment could occur for size (unknown annual growth increment), year comparisons were limited to 2014–2015 and 2015–2016, assuming only a small incremental increase in size between these periods. To evaluate possible bias associated with combing fall and spring samples, each sample of spring-tagged fish was analyzed separately for size and age-thresholds.

### Migration pathways

Egress pathway from the Chesapeake Bay to ocean waters was evaluated by detection of emigrants by receivers immediately inside or outside the mouth of the Chesapeake Bay and others occurring in the Chesapeake and Delaware (C&D) Canal. The C&D Canal is < 1 km in width and is well covered by the 2–3 receivers that occur in the Canal in a given year (receiver detection range is ~0.6–1.0 km [27]). On the other hand, the Chesapeake Bay mouth is incompletely covered with respect to number of receivers and their expected detection ranges. Patterns of seasonal ocean migration were evaluated by depicting seasonal progression of detections at receivers ordered by latitude and region. Individual detections were assigned to shelf regions to depict seasonal shelf migration pathways. Shelf regions were assigned to represent clusters of receivers over the shelf and include New York (NY Harbor and its approaches), Long Island Sound, and coastal Massachusetts, New Jersey, Delaware, Maryland, and Virginia.

### Skipping and straying

For fully mature individuals (>80 cm TL), evidence of spawning site fidelity was on the basis of 15 March– 15 May detections by receivers at or above the Potomac River Nice Bridge, which occurs just downriver from spawning habitats [34]. Evidence for non-natal spawning in the Hudson or Delaware Rivers followed conventions for seasonal occurrence: Delaware, above river km 50, 15 March-15 May; Hudson above river km 60, 15 April-15 June [14]. Non-natal spawning in other tributaries of the Chesapeake Bay was classified based on incidence in mid- or upper estuarine locations during the 15 March—15 May period. Non-annual (“skipped”) spawning was assigned to those ocean-classified fish that did not enter spawning estuaries during 15 March-15 June, under the assumption that all spawning estuaries in the Chesapeake Bay and the Delaware and Hudson estuaries had full telemetry coverage (Fig 1).

## Results

### Differential mortality

**Fig 3:**
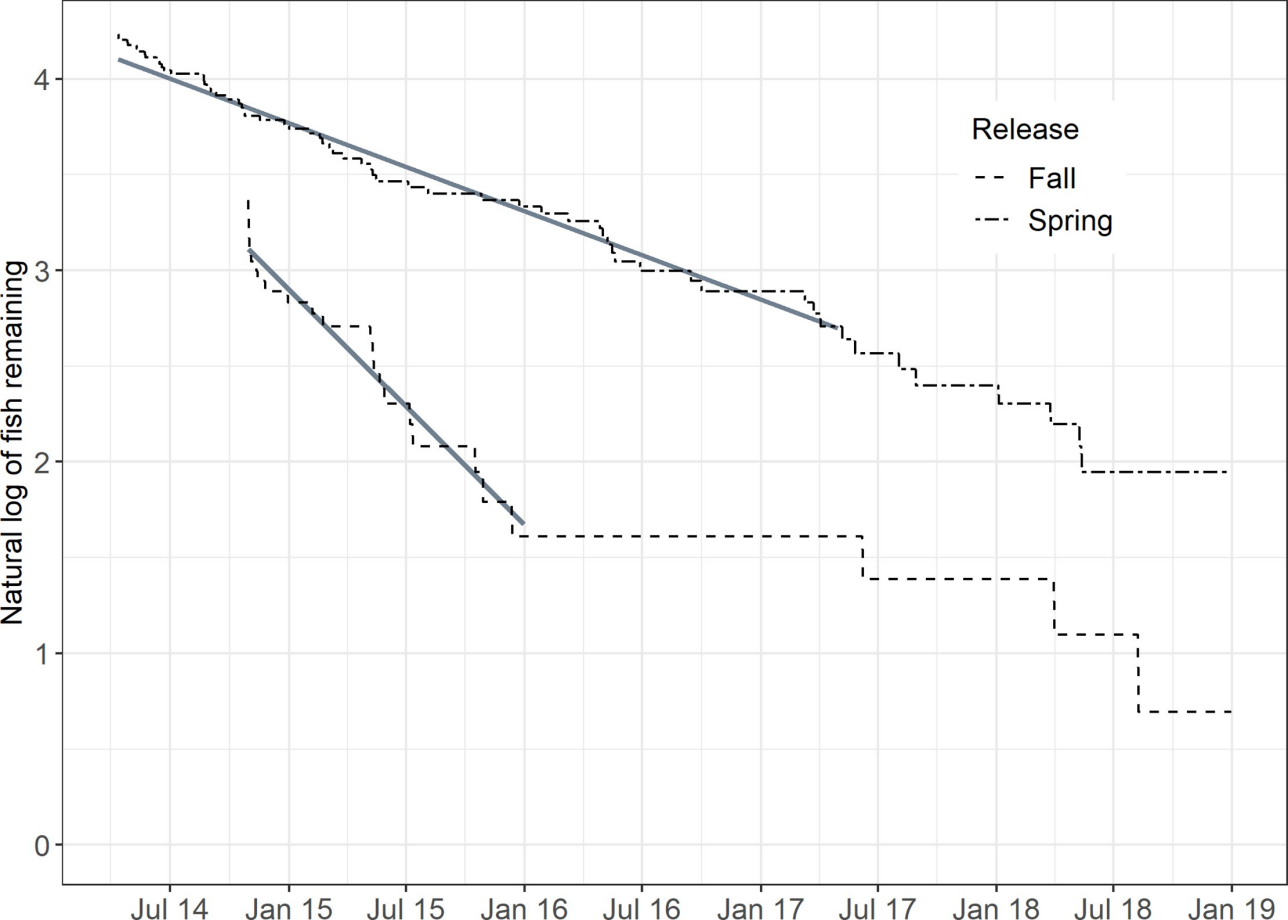
Attrition (log_e_ n) of spring and fall released Potomac River striped bass. Fitted regression lines are overlain.

The spring release was detected over a >4-yr period and experienced an exponential rate of loss over the first three years of the manufacturer-specified tag lifespan (Fig 3; R^2^ = 0.98; n = 1116 interpolated daily estimates; p<0.001). Negative residuals indicated slightly higher loss rates during springs of 2015 and 2016 in comparison to other seasonal periods. Rate of loss was much higher for fall releases, also showing an exponential rate of decline (Fig 3; R^2^ = 0.96; n = 428 daily estimates; p<0.001). At 1.5 years post-release, only five of 25 fall-released fish remained in the study, curtailing further analysis. Elevated periods of loss included, (1) the 2 weeks immediately after the fall release, (2) May-July 2015, and (3) October-December 2015. Instantaneous loss rates were -1.27 10^−3^ (95% CI: -1.28 10−3 –-1.26 10^−3^) and -3.32 10^−3^ (95% CI: -3.39 10−3 –-3.26 10^−3^) for the spring- and fall-release groups, respectively. These daily rates translate to annual rates of -0.46 (36.9% yr^-1^; 2014–2019) and -1.21 (70.3% yr^-1^; 2014–2016) for the spring- and fall-release groups, respectively.

### Differential migration

**Fig 4:**
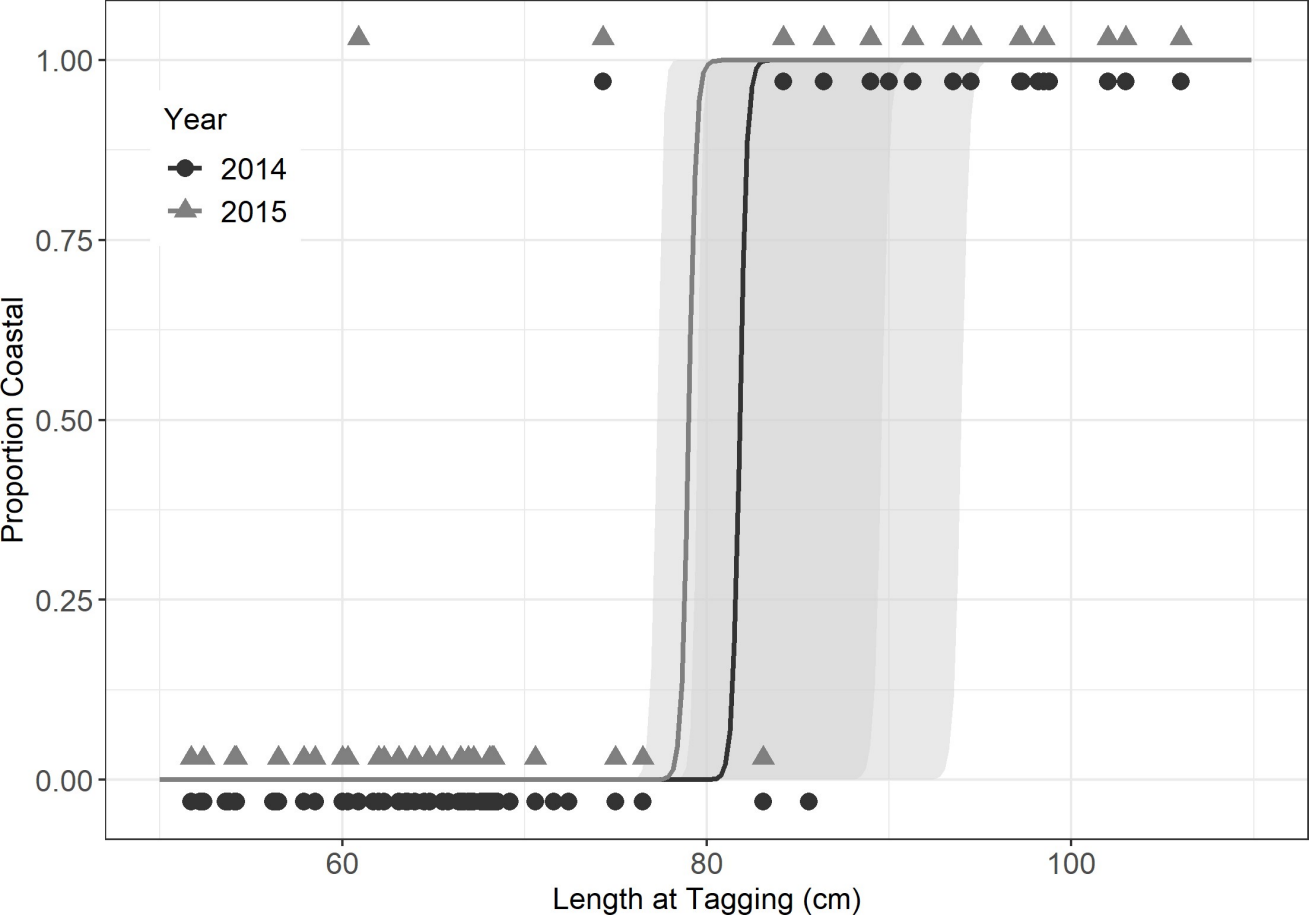
Size-dependent emigration by study striped bass. GLMM fits of ocean incidences versus total length for striped bass classified based on positive or nil detections in shelf arrays. 95% confidence interval ellipses are shown.

**Fig 5:**
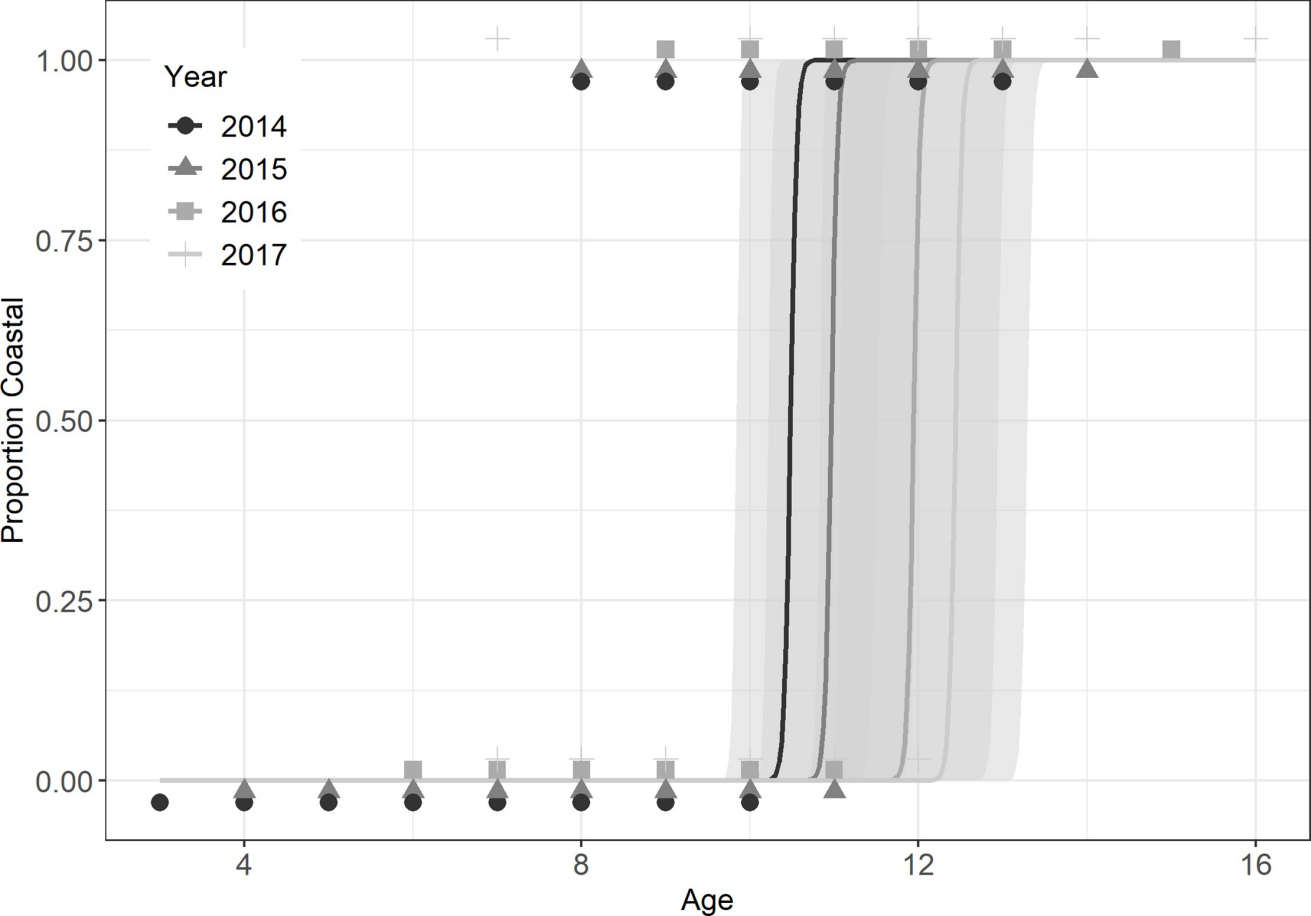
Age-dependent emigration by study striped bass. GLMM fits of ocean incidences versus estimated age for striped bass classified based on positive or nil detections in shelf arrays. 95% confidence interval ellipses are shown.

Ocean emigration, classified based on positive detections in shelf arrays, was well fit by logistic functions of TL (Fig 4), and age (Fig 5) (weight not shown). Logistic coefficients were significant at alpha = 0.05 for all fitted GLMM models. For TL_50_ models, the intercept but not slope of the year effect was significant. For the first year after release, ocean incidence rose sharply at sizes greater than 80 cm TL, with an estimated TL_50_ = 81.9 cm (Fig 4; Table 1). The second year’s TL_50_ estimate, adjusted for the year effect, yielded a similar TL_50_ estimate (79.0cm). Estimates for Age_50_ ranged from 10.5 to 12.4 across years (Fig 5). Here, age at release was adjusted for each sequential year analyzed, limiting systemic bias, and no year effect was detected in the GLMM model. Still, Age50 increased from 10.5 to 12.4 years in an ordered fashion according to year of detection (Fig 5). The fall sample was fully resident (data not shown). Separate analysis of the spring sample resulted in similar size and age thresholds (Table 1).

**Table 1:** Estimates of 50th percentile shelf migrant incidence for length, weight and age.

		TL_50_^a^			Wt_50_^b^			Age_50_^c^		
Year	n	LCI^d^	Estimate	UCI^e^	LCI	Estimate	UCI	LCI	Estimate	UCI
Combined Fall- and Spring-tagged samples										
**2014**	69	72.1	81.8	93.1	3.7	6.5	9.1	8.6	10.5	12.4
**2015**	42	70.8	79.0	88.9	2.9	6.1	8.3	9.5	11.0	12.5
**2016**	25	-	-	-	-	-	-	10.5	11.9	13.4
**2017**	17	-	-	-	-	-	-	10.7	12.4	14.1
**Overall**		71.5	80.4	90.6	3.3	6.3	8.8	10.0	11.5	13.0
Spring-tagged samples only										
**2014**	57	70.7	81.8	92.9	3.8	6.5	9.0	8.8	9.7	10.9
**2015**	32	61.1	79.0	88.8	3.7	6.1	8.2	9.2	10.3	11.3
**2016**	20	-	-	-	-	-	-	10.1	11.3	12.3
**2017**	13	-	-	-	-	-	-	11.0	12.3	13.3
**Overall**		69.8	80.4	90.4	3.8	6.3	8.6	9.8	10.9	11.9

^a^TL_50_ = 50th percentile total length (cm)

^b^Wt_50_ = 50th percentile weight (kg)

^c^Age_50_ = 50th percentile age (yr), adjusted for annual age increments across years

^d^LCI = lower 95% confidence estimate

^e^UCI = upper 95% confidence estimate

The majority of ocean migrants were female, but this reflected the female bias within the tagged sample of large fish. Of the four large males (>80 cm TL), one failed to meet the inclusion rule (successive spring detections) and the other 3 were classified as ocean migrants during 2014.

### Migration pathways

Ocean emigrants from the 2014 spring-release increased from 29% in 2014 to 42% in 2016, likely owing to growth of larger resident individuals in 2014 into sizes more likely to emigrate during 2015 and 2016 (Table 2). Of those fish classified as emigrants and detected using one of the Chesapeake Bay’s exit corridors, the majority (80%; 37/46) departed through the mouth of the Chesapeake Bay for the period 2014–2017. For an unknown reason in 2014, the majority of emigrants (65%; 13/20) was not detected as they emigrated from the Chesapeake Bay, despite similar telemetry receiver deployments for the period 2014–2017.

**Table 2:** Numbers and percentages of estimated emigrants according to exit routes from the Chesapeake Bay.

Year	Total Detections^a^	Emigrants	Exit: C&D Canal	Exit: Bay Mouth	Exit: Unknown
**2014**	70	20	2 (10%)	5 (25%)	13 (65%)
**2015**	59	19	7 (37%)	12 (63%)	0
**2016**	33	14	0	14 (100%)	0
**2017**	23	9	0	6	3

**Fig 6:**
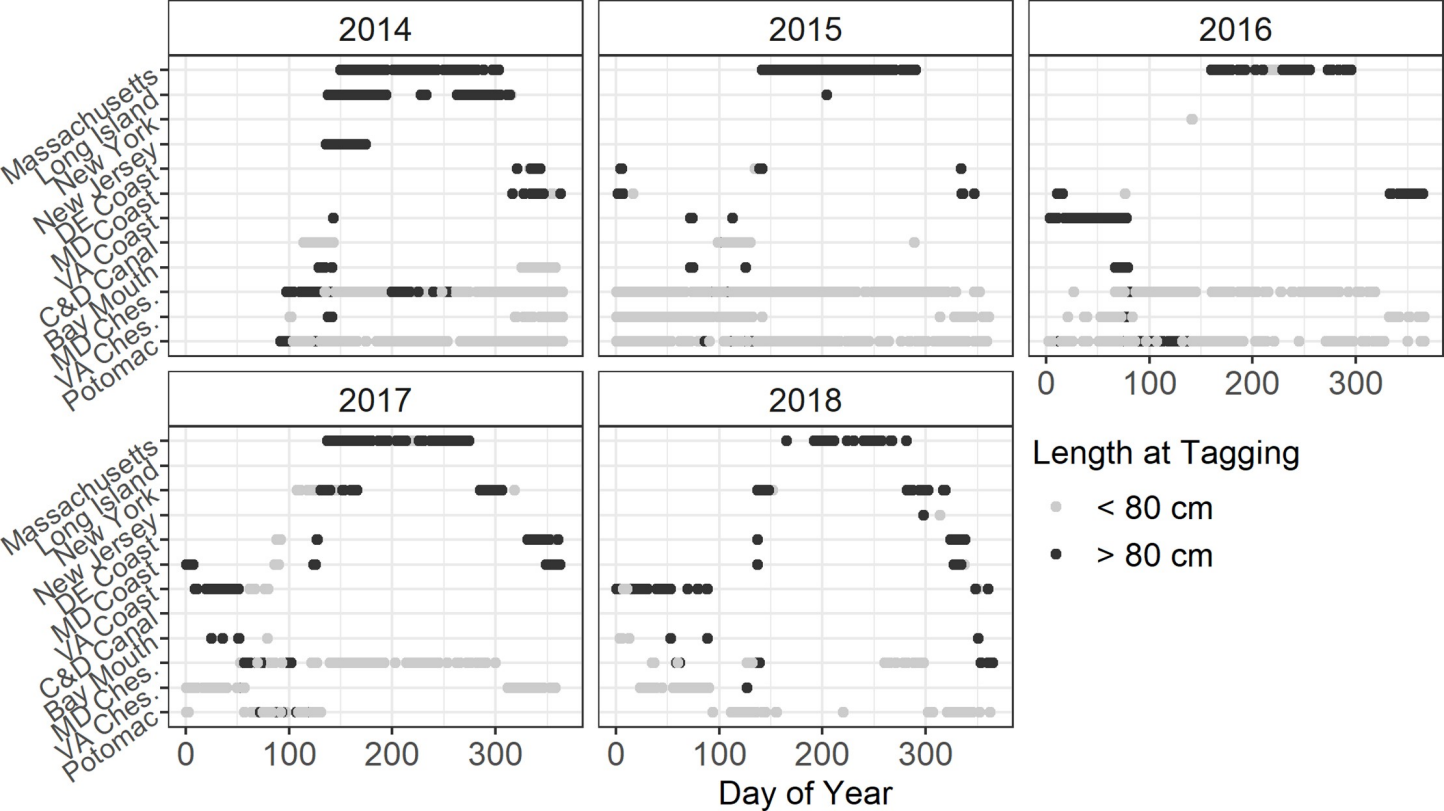
Seasonal migrations by study striped bass in the Chesapeake and NW Atlantic shelf waters. Symbols indicate daily detections for small (≤80 cm TL) and large (>80 cm TL) size classes. Locations are arranged along the y-axis according to latitude.

The majority of large (>80 cm TL) ocean striped bass arrived into Massachusetts waters within 60 d after their release (Fig 6). Although a small fraction lingered for a 60 d period in waters off Long Island, all ocean fish eventually moved into Massachusetts waters, where they persisted from June through October (a single fish persisted until January 2015), with a small group departing in August again lingering off Long Island. Southward migrations occurred during November-January for the 2014 release group. Loss of NY Bight receivers in 2015–2017 curtailed evaluation of seasonal migrations. Still, the sequential progression of rapid spring ocean migration over a 60 d period, to extended summertime occurrence in Massachusetts water, to a slower southward migration (~90 day period) held for each year. Note the lack of summertime incidence off DelMarVa. In contrast, this area appears to support winter occurrences, particularly evident in 2016, when the US Navy array substantially expanded in shelf regions adjacent to the mouth of the Chesapeake Bay (Fig 1). Increased ocean incidence of fish released at <80 cm TL occurred with each successive year as these fish grew into the larger size class predicted to migrate (Fig 6).

Nearly all implanted fish ≤80 cm TL remained in the Chesapeake Bay throughout the year in all study years (Fig 6). Year-round residency was observed in the Potomac River and the upper portion of the Chesapeake Bay, but was not observed for the lower portion of the Chesapeake Bay. A minority of larger fish also persisted in the Chesapeake Bay in each year.

### Straying and skipping

We observed spawning site fidelity (annual spring return to the Potomac River) in the majority of large individuals (Table 3), with straying only observed to occur in other Chesapeake Bay tributaries. Estimated skipped spawning was substantially higher the first year after release than in subsequent years.

**Table 3:** Spawning site fidelity, straying and skipped spawning by study striped bass.

	TL≤80 cm		TL>80 cm					
						Straying		
	N	Spawn Fidelity	N	Spawn Fidelity	Skipped Spawning	Delaware Estuary	Hudson Estuary	Other Chesapeake tributary
**2015**	37	15	14	8	5	0	0	1
**2016**	18	8	13	10	2	0	0	1
**2017**	15	8	7	6	1	0	0	0

## Discussion

### Differential migration

The three- year biotelemetry study on Chesapeake Bay striped bass gave clear evidence of differential migration. Study striped bass emigrated at sizes greater than the 50^th^ percentile threshold size of 80 cm TL (referenced as >80 cm TL in classifications used in Results). At these lengths, all males and most females are sexually mature [35] and fish dominate the diet [36]. Locomotion costs decrease with fish size, increasing the capacity to exploit distant foraging habitats [9]. As fish migrate from natal waters, larger and farther ranging individuals may exhibit increased propensity to stray or skip spawning [37–39]. We observed the opposite (Table 3), which could relate to learned migration circuits that may become increasingly rigid as they are repeated each year by older individuals. This speculation is supported by the rapid early summer transit to Massachusetts shelf waters by all emigrating Potomac striped bass. Strong inter-annual patterns of site fidelity to localized regions with Cape Cod and Massachusetts Bays, reported by Kneebone et al. [14], might also be achieved through learned migration behaviors. Additionally, smaller individuals were predominantly males, known to exhibit higher straying rates than females [40, 41].

To an unexpected degree, our threshold size (80 cm TL) and age (10 years) closely agreed with those documented in past studies (80 cm TL [25] and 10–12 years [23]); we had expected that telemetry of a moderately large sample would expose greater variance in movement behaviors than these past studies. In Dorazio et al.’s analysis of tag recaptures, reporting rates were quite low (<5%; recapture sample = 56), likely introducing substantial error owing to reporting bias and low sample size. Secor and Piccoli back-calculated size at egress from otolith microchemical assays of large striped bass collected from the Northern Bay mainstem and Choptank River (Chesapeake Bay; n = 122), and observed that 50% emigrated by ages 10–12, similar to that observed here. On the other hand, a substantial fraction of fish >10 years old were estimated to remain in the Chesapeake Bay. Still, the 29 salinity unit threshold used to distinguish oceanic from estuarine incidence by Secor and Piccoli is not mirrored by a threshold change in otolith Sr:Ca. Thus, threshold sizes and ages for egress are less well supported though otolith chemistry Sr:Ca than other approaches such as biotelemetry.

Biotelemetry research associated with the other two principal populations, the Hudson and Delaware Rivers, support similar size ranges for migratory ocean fish. All but one of 20 spawning-run Hudson River striped bass (82–97 cm TL) migrated during summer months to Massachusetts waters [26]. The exceptional fish remained resident to the Hudson River estuary during the two-year study. Indeed, lifetime patterns of residency are well documented for Hudson River striped bass [42, 43]. Size-dependent migrations for the Delaware population have not yet been specifically assessed, but a moderate fraction (12–108) of large striped bass (65–110 cm TL, mean = 88±11 cm) tagged and released in Massachusetts waters visited the Delaware Bay during the spawning season [14].

Our results did not support Kohlenstein’s early hypothesis that there is a large pulse of females emigrating at 3 years of age and males remain resident throughout their lives [24]. Here we have shown that very few fish are destined to be resident should they grow into size classes >80 cm TL. Further, multiple studies and approaches now confirm that striped bass are substantially older than 3 years at emigration and, although ocean migrants are dominated by females, this relates to their predominance among large size-classes rather than, say, sex-specific behaviors within size classes. Past studies [23, 25] have also failed to detect sex-specific differences in emigration after controlling for size.

### Differential mortality

Striped bass that remained in the Chesapeake Bay had higher mortality rates than those that undertook ocean migrations. The assumption that fall-tagged and released striped bass were resident to the Chesapeake Bay was confirmed through telemetry and conformed to predictions of size-specific emigration (Fig 4). Their small size and age at tagging, indicated over the 1.5 year period that losses were assessed, ocean emigration was unlikely. Although the spring spawning run sample included resident individuals (i.e., those ≤ 80 cm TL), a large fraction undertook ocean migrations following release with individuals continuing to recruit to the ocean contingent as they grew. Under the limits of this assumption, the spring-sample mortality rate is likely biased high as it contained some resident individuals. Thus, the nearly two-fold higher mortality rate of the resident group (70.3 36.9% yr^-1^) in comparison to the presumed ocean contingent (36.9% yr^-1^) is likely conservative.

### Other migration behaviors

Differential migration was the dominant, but not exclusive, form of migration plasticity exhibited by study striped bass. A very small minority of >80 cm striped bass (n = 2) resided during summer months in the Chesapeake Bay. The Potomac River population is quite large with the potential for multiple spawning runs [44], trophic partitioning [45], and other contingent behaviors that may have not been well represented. Another migration behavior not observed here but well documented is the occurrence of age 1–3 striped bass in shelf waters [13, 15]. Recent telemetry work has focused on aggregations of these small fish in the shelf waters off NJ and Southern New England [20, 21, 46, 47], but because they were not subsequently tracked during spawning tributaries, inferences on where they came from are speculative [48]. Early research [13] noted high abundances of small striped bass in Southern New England waters and attributed them to strong recruitment years in the Chesapeake Bay. The provenance of young ocean striped bass—whether regional spill over from natal systems or the result of longer ocean migrations, remains unknown–a topic that would be well engaged through genetic markers or additional telemetry research. Additionally, large aggregations traditionally fished within the nearshore 5-km US state jurisdictional waters [49, 50] have apparently shifted to offshore federal waters causing increased allocation and enforcement challenges [51].

### Migration pathways

Biotelemetry has allowed us to move beyond the question of whether Potomac River striped bass leave the Chesapeake Bay [16, 24, 52], to where do they go when they leave? All arrows point to Massachusetts. The strong connectivity between large Potomac River spawners and feeding aggregations in nearshore waters off Cape Cod and in Massachusetts Bay is remarkable given that 100% of emigrants wind up here after a rapid directional summer transit. Transit times ranged from 17 to 96 days with a mean of 47 days during springs 2014–2018. A complementary biotelemetry study [14] implanted large striped bass in Massachusetts waters and found similar southerly (winter) and northerly (late spring) shelf occurrences in shelf telemetry arrays. Unfortunately, the Chesapeake Bay and southern Mid Atlantic contained few telemetry assets during their study. Still, their conclusion that most of their implanted striped bass originated from Chesapeake waters is well supported by our study.

Winter migrations were also rapid (Fig 6), but the final endpoint in ocean waters (i.e., overwinter habitat) is less certain than the Massachusetts summer feeding habitat. With the deployment of large shelf arrays in DelMarVa near-shelf waters (Fig 1: 2016–2018), winter habitats were brought into clearer view. VA shelf waters showed high January-February incidence, and during the winter 2016–2017, a warmer year [53];, high December-January incidences also occurred in Maryland shelf waters. Still, the lack of telemetry assets in North Carolina (NC) shelf waters, a known wintering area for striped bass [54, 55], limit inferences of where Potomac striped bass overwinter. Past genetics [56] and conventional tagging data [54] indicate that striped bass from the Chesapeake Bay in recent times have utilized the NC overwintering area. Continued warming of Mid-Atlantic shelf waters [57, 58] could result in greater overwintering off DelMarVa. Still, the NC shelf is also a key wintering-foraging habitat for other oceanic migrating species including sharks, bluefish, weakfish, menhaden, and tunas [59–63] and merits priority in future telemetry studies.

### Straying and skipping

A fraction of large striped bass were not detected in the Potomac River during the spawning period each year, exhibiting so-called skipped spawning. Skipped spawning (or alternatively, mis-specified reproductive schedules, [64]) can occur owing to energetic constraints and seasonal movements that bypass spawning run behaviors [65]. A much higher level of skipped spawning was observed during the first spring after release (2015; 36%) than the subsequent two years (2016–2017; 14–15%). This could suggest a latent effect of capture, surgery, and release affecting reproduction the subsequent year. On the other hand, we did not observe a similar effect in a past study on spawning run Hudson River striped bass [26]. Further investigation is warranted to limit telemetry study impacts on subsequent condition and reproduction of large striped bass [66].

Straying, incidence during spring in non-natal tributaries, was quite rare for large striped bass. No large fish strayed to tributaries outside the Chesapeake Bay and only two visited non-natal tributaries within the Chesapeake Bay (the adjacent Patuxent and Rappahannock Rivers). These two individuals were detected in spawning areas within the Hudson River during spring. Genetic separation between Hudson and Chesapeake Bay populations [41, 67] suggests that such straying may be uncommon. An interesting possibility, known as adopted migration [6, 68, 69] is that these new ocean emigrants may have been susceptible to non-natal migration circuits, adopting those of Hudson River striped bass through population overlap and social interactions in shelf waters.

### Management implications of differential migration

Ocean emigration by striped bass continues to challenge fishers in their pursuit and fishery managers in their assessment of how source populations contribute to shelf fisheries. Ocean fisheries are assessed and managed by the Atlantic States Marine Fisheries Commission (ASMFC) using a Statistical Catch at Age model comprised of two “fleets,” one focused on the commercial fisheries of the Chesapeake Bay and the second on recreational ocean fisheries. Consideration of fleet behaviors is important owing to differing selectivity patterns between recreational (selection for large sizes) and commercial (selection for intermediate sizes) fisheries. Further specification of population-specific behaviors by Chesapeake Bay striped bass was pursued in a two-stock model (Chesapeake Bay population v. everything else) in the most recent assessment [12] but did not pass peer review, owing in part to uncertainty in the population’s contribution to ocean fisheries and whether size at emigration has changed through time [70]. Because we observed similar TL_50_ levels as past studies by Dorazio et al. [25] and Secor and Piccoli [23], we infer that size at emigration for Chesapeake Bay striped bass has been stable in recent decades. This supports a key assumption of the ASMFC’s spatially explicit stock assessment, one that specifies differential migration and holds considerable advantage in specifying the productivity of estuarine and ocean segments of the fishery to better support regional allocation tactics.

Emigration by striped bass, referenced here as differential migration, represents a type of partial migration, influencing population production, resilience, and stability. Spatial buffering against regional differences in exploitation, pollution, and food web conditions can occur when contingents within populations vary in their migration patterns as documented here for striped bass. Indeed, the dramatic recovery of striped bass in the 1990s was largely due to differential migration, which allowed escapement of large ocean striped bass (i.e., facilitated differential migration) from extremely intense fisheries on smaller individuals within the Chesapeake Bay [71]. Thus, partial migration controls have been implicit in past key management tactics. Further, differential migration by striped bass defines the role that they play in estuarine and shelf food webs as forage demands are strongly size-dependent. For the year 2017, the ASMFC assessed a high total natural mortality rate: 64% for resident Chesapeake Bay striped bass based on a tagging study [12]. This mortality was attributed to disease (*Mycobacteriosis spp*.) prevalence, although estimates of fishing mortality are uncertain owing to hook and release mortality [72]. The assessed mortality level is similar to transmitter loss observed in our study (70% yr^-1^). Total mortality for the ocean contingent of Chesapeake Bay striped bass estimated by the ASMFC was 36% yr^-1^, which was also similar to transmitter loss levels for this population segment (40%). Thus, the capacity of differential migration to contribute to stability in the overall population and to convey key trophic roles across its range will have much to do with managing exploitation and other sources of mortality in both the Chesapeake Bay and shelf regions.
